# COVID-19 Pandemic in the Italian Population: Validation of a Post-Traumatic Stress Disorder Questionnaire and Prevalence of PTSD Symptomatology

**DOI:** 10.3390/ijerph17114151

**Published:** 2020-06-10

**Authors:** Giuseppe Forte, Francesca Favieri, Renata Tambelli, Maria Casagrande

**Affiliations:** 1 Department of Psychology, Sapienza University of Rome, Via dei Marsi 78, 00185 Rome, Italy; francesca.favieri@uniroma1.it; 2 Department of Dynamic and Clinical Psychology, Sapienza University of Rome, Via degli Apuli 1, 00185 Rome, Italy; renata.tambelli@uniroma1.it

**Keywords:** COVID-19, pandemic, post-traumatic stress disorder, PTSD, prevalence

## Abstract

Since December 2019, the COVID-19 pandemic has attracted worldwide attention for its rapid and exponential diffusion. The long-term psychological impact, of both the spread of the virus and the restrictive policies adopted to counteract it, remains uncertain. However, recent studies reported a high level of psychological distress and Post-Traumatic Stress Disorder (PTSD) symptoms. The purpose of this study is to assess the psychometric properties of a new questionnaire, to evaluate PTSD risk related to the COVID-19 emergency. A total of Italian people completed a web-based cross-sectional survey broadcasted through different social-media. Demographic data and some psychological dimensions, such as general distress and sleep disturbance, were collected. A new self-report questionnaire (COVID-19-PTSD), consisting of 19 items, was developed starting from the PTSD Check List for DSM-5 (PCL-5) questionnaire, and it was administered in order to analyze its psychometric properties. The results highlighted the adequate psychometric properties of the COVID-19-PTSD questionnaire. The confirmatory factor analysis indicated that a seven-factor model (*Intrusion*, *Avoidance*, *Negative Affect*, *Anhedonia*, *Dysphoric arousal*, *Anxious arousal* and *Externalizing behavior)* best fits the data. Significant correlations were found among COVID-19-PTSD scores, general distress and sleep disturbance. A high percentage of PTSD symptomatology (29.5%) was found in the Italian population. COVID-19-PTSD appears to be effective in evaluating the specific stress symptoms related to the COVID-19 pandemic in the Italian population. These results are relevant from a clinical point of view because they suggest that the COVID-19 pandemic could be considered as a traumatic event. Psychological interventions to counteract short- and long-term psychopathological effects, consequent to the COVID-19 pandemic, appear to be necessary.

## 1. Introduction

Since December 2019, an outbreak of pneumonia associated with a new coronavirus (i.e., SARS-CoV-2; COVID-19) has been reported in several parts of the world, and its rapid and exponential diffusion forced the World Health Organization (WHO) to declare it a pandemic [1]. Social distancing, confinement and quarantine were adopted by many countries to contain the diffusion of the infection. 

As reported by some studies [2,3,4,5,6], these extreme measures, along with the fear and uncertainty of an unknown infection, have impacted on people’s lifestyles, generating high levels of psychological distress, anxiety and mood alterations. Both the fear of contracting the virus and the measures adopted to counteract the spread of infection may have been perceived as traumatic events [7]. Consequently, they can represent risk factors for many mental diseases [8], and they can potentially generate Post-Traumatic Stress Disorder (PTSD) symptoms [8]. 

PTSD is a psychiatric disorder caused by a terrifying event, perceived as a trauma, which affects directly or indirectly the individual (e.g., severe accident or injury, threat to physical safety, death or threat death, sexual assault, natural disasters, war, etc.) [9]. Specific criteria, focused on identifying causes and symptoms, are required for the PTSD diagnosis. The identification of the stressor is a first, but not sufficient, criterion for the PTSD diagnosis (Criterion A), followed by the identification of symptoms related to four specific dimensions (B, C, D, E Criteria): intrusive symptoms; avoidance; negative alterations in mood and thinking; changes in arousal and reactivity [9]. The negative impact of the PTSD symptomatology on daily life (e.g., social interaction, work activity) is another criterion to consider for its functional significance (Criterion G) [9]. 

The pandemic outbreak of an unrecognized infection, with no vaccines or effective medical treatments, such as COVID-19, could be defined as a traumatic experience for its acute and chronic implications at individual and community levels. The COVID-19 outbreak has had a direct effect on the population. On the one hand, the fear of contagion and the risk of death, for oneself and loved ones, represents a direct threat (Criterion A of Diagnostic and Statistical Manual of Mental Disorders, Fifth Edition; DSM-5). On the other hand, indirect consequences of the pandemic appear to be associated with feelings of instability, psychological distress, sleep disturbance, psychiatric and mood disorders, and general psychopathological symptomatology [2,3,4,5,6,8,9,10]. These symptoms may be due to, or occur in comorbidity with, those of the DSM-5 criteria for the PTSD diagnosis.

As evidenced by previous studies, PTSD is reported in epidemics or other medical emergencies [11,12,13,14] in the months following the critical period. Moreover, PTSD symptoms were reported in the first studies of the COVID-19 emergency in China and Italy [5,6,15]. A high risk of developing PTSD was evidenced in: (a) survivors who faced the threat of death; (b) healthcare workers, who were overworked with limited safety equipment, and who experienced feelings of powerlessness and helplessness; (c) healthy people who have had direct contacts with the infection, experiencing fear related to the risk of infection; (d) the general population subjected to restrictive measures (e.g., social distancing, quarantine, isolation), and overexposure to media information about the pandemic. All these aspects can generate feelings of isolation, frustration and anticipatory anxiety. 

Considering the unique characteristics of the COVID-19 emergency, which could generate a novel perspective on trauma, the present study aims to develop a new questionnaire to assess PTSD symptomatology, related to the COVID-19 pandemic. The COVID-19-PTSD questionnaire, following the items of the PTSD Checklist for the DSM-5 (PCL-5) [16], is focused on direct (e.g., fear of the infection) and indirect (e.g., social distancing, social isolation, quarantine) stressors consequent to the COVID-19 emergency. Given the similar factors that characterize all medical emergencies, this questionnaire could be useful also in the future, in order to define the PTSD symptoms associated with them.

The objectives of this study are manifold. The first aim is to assess the validity and reliability of the COVID-19-PTSD questionnaire in a sample of Italian people, testing its factorial structure (one, four, or seven factors) with a confirmatory approach. The second purpose is to evaluate its internal consistency. The third is to examine both convergent and discriminant validity, by comparing the results of the COVID-19-PTSD questionnaire with those obtained from the Impact of Event Scale—Revised (IES—R) [17], one of the most widely-used self-report measure for assessing individual stress response to a traumatic event, also used in the Italian population [18,19,20,21,22,23,24,25]), where a validated version of PCL-5 is not available. Furthermore, given the high comorbidity of PTSD with anxiety, depression, distress and sleep disturbances, it has also been formulated based on the moderate to high positive correlations between the Symptom Checklist-90 (SCL-90) [26] and the Pittsburgh Sleep Quality Index [27] scores, and the COVID-19-PTSD-related symptoms. The fourth aim is to estimate the prevalence of PTSD symptoms in an Italian sample after the first weeks of the COVID-19 outbreak and the subsequent lockdown.

## 2. Method

### 2.1. Participants

A total of 2286 respondents participated in the study. Of the total respondents that started the questionnaires, 98% (2286 out of 2332 people) completed the whole survey, and were considered for the statistical analyses. There were 1706 women (74% of the sample). The mean age of the participants was 29.61 (SD = 11.42), and the age ranged between 18 and 74 years. 

### 2.2. Questionnaires 

#### 2.2.1. Demographic Questionnaire and COVID Related Information

The first section of the survey collected information on demographic variables, including sex (men or women), age, education and occupation, city and region of origin, and medical and psychopathological history. The second section assessed personal experience related to the COVID-19 outbreak.

#### 2.2.2. Post-Traumatic Stress Disorder Related to COVID-19 Questionnaire (COVID-19-PTSD) 

The COVID-19-PTSD is a self-report measure designed ad hoc to assess specific symptoms concerning the risk of PTSD in the actual pandemic emergency. All questions refer to the previous seven days during the COVID-19 outbreak. 

A preliminary set of items was first created, starting from the 20 items of PCL-5 corresponding to the criteria for PTSD outlined in the DSM-5. Then, a focus group, to improve content validity, was conducted by the last author (MC), with five clinical psychologists with expertise in the DSM-5 criteria for PTSD diagnosis. The group tried to identify central and specific traumatic aspects of pandemic exposure. This procedure allowed the suggestion of some modifications to the items of the PCL-5. These adjustments were discussed and evaluated by all the group, and eligible items were selected by agreement among participants. Any disagreement was discussed with the supervisor. 

The items of PCL-5 were modified in order to focus the attention on a prolonged and current stressor. Two items of the PCL-5 were deleted [“Suddenly feeling or acting as if the stressful experience were actually happening again (as if you were actually back there reliving it)?”; “Trouble remembering important parts of the stressful experience?”], because they assessed areas already measured by other items (Intrusion and Avoidance); furthermore, they were found to specifically evaluate the effects of an acute trauma, rather than the exposure to prolonged trauma, such as the COVID-19 emergency. Finally, because the sleep disturbance was identified as a recurrent symptom in the COVID-19 emergency [5,6,15], we added another item to evaluate sleep quality (i.e., To have a disturbed sleep). It was added to the two items already present (i.e., Repeated and disturbing dreams about this stressful experience; Having trouble falling asleep). 

The final version of the questionnaire includes 19 items, requiring a response on a 5-point Likert scale, from 0 (not at all) to 4 (extremely). The instructions provided to the respondents are: “Referring to the current situation, characterized by the COVID-19 outbreak and the social distancing measures implemented to contain it, indicate how you feel for each of the following dimensions” (see Table S1). 

To assess the factorial structure of the COVID-19-PTSD questionnaire, we tested three hypotheses. Firstly, based on our previous study [10], we verified a monofactorial structure. Secondly, according to DSM-5, we tested the four-factors structure that would correspond to the four-symptom clusters, i.e., *Re-experiencing*, *Avoidance, Negative alterations in cognition and mood*, and *Increased arousal and reactivity.* Thirdly, in line with the study of Ashbaugh et al. [28], a seven-factors structure (*Intrusion*, *Avoidance*, *Negative Affect*, *Anhedonia*, *Dysphoric arousal*, *Anxious arousal*, and *Externalizing behavior*) was examined. 

As far as we know, this is the first questionnaire that assesses PTSD symptoms in a protracted stressful situation that depends on a severe outbreak. Therefore, it may be useful to test multiple factorial models to verify which of these best fits the data related to the PTSD symptomatology. 

#### 2.2.3. Impact of Event Scale—Revised (IES—R)

IES—R [17,25] is a self-report questionnaire that assesses subjective distress for different specific life events, according to the Diagnostic and Statistical Manual of Mental Disorders, Fourth Edition (DSM-IV) criteria for PTSD. It includes 22 items, requiring a response on a 5-point Likert scale (from 0 = not at all, to 4 = extremely). The IES—R measures PTSD symptoms severity in the past seven days, with three subscales assessing *Avoidance* (the tendency to avoid thoughts or reminders about the incident), *Intrusion* (difficulty in staying asleep, dissociative experiencing, similar to flashbacks), and *Hyperarousal* (irritated feeling, angry, difficulty in sleep onset). In addition to the three subscale scores, IES—R also gives an overall score of events impact (IES—R total, equal to the sum of the three subscale scores). The cut-off of 33 was adopted to indicate a high risk of PTSD symptomatology, in line with the literature. The Italian translation of the IES—R showed satisfactory internal consistency in studies on different at-risk populations, as reported by Craparo et al. [21] (Intrusion, α = 0.78; Avoidance, α = 0.72; Hyperarousal, α = 0.83) and Converso and Viotti [24] (Intrusion, α = 0.91; Avoidance, α = 0.81; Hyperarousal, α = 0.87). 

Several factors directed the choice to use the IES—R to evaluate the convergent validity of the COVID-19-PTSD. First of all, although the IES—R has not been validated in the general Italian population, it was used to assess the PTSD symptomatology in many Italian samples [18,19,20,21,22,23,24,29,30]), which confirmed its adequate reliability. Moreover, both a validation of the previous version (IES) [31] and a published translation of the current version (IES—R) [26] led us to consider the IES—R as an appropriate tool to use for assessing the convergent validity of the COVID-19-PTSD questionnaire. Finally, the absence of an Italian version of the original PCL-5 supported this choice.

#### 2.2.4. Symptom Checklist—90 (SCL-90)

SCL-90 [26,32] assesses psychological distress and symptomatology. It includes 90 items, rated on five-point Likert scales, ranging from ‘not at all’ (0) to ‘extremely’ (4). The psychopathological dimensions assessed are Somatization, Obsessive-Compulsive, Interpersonal Sensitivity, Depression, Anxiety, Anger-Hostility, Phobic Anxiety, Paranoid Ideation and Psychoticism. A Global Severity Index provides a measure of the overall psychological distress. Higher scores in each dimension indicate greater distress and psychopathological symptomatology. Internal consistency of SCL-90 was good for all subscales (α values ranging between 0.70 and 0.96). 

#### 2.2.5. Pittsburgh Sleep Quality Index (PSQI)

The PSQI [27,33] is a 19-items questionnaire used to evaluate sleep quality, sleep duration, sleep latency, habitual sleep efficiency, sleep disorders, the use of sleeping medications and daytime dysfunctions. Each dimension scored between 0 and 3, with a total score ranging from 0 to 21. Higher scores indicate lower sleep quality. The Italian version of PSQI showed a high internal consistency (Cronbach’s α = 0.835).

#### 2.2.6. State-Trait Anxiety Inventory (STAI-Y)

The STAI-Y [34,35] is a 40-items questionnaire, structured on 4-point Likert scales (from 0 = not at all, to 4 = extremely). It measures state (STAI-S) and trait (STAI-T) anxiety, i.e., how participants feel about anxiety “now, at this moment”, and how they “generally feel” about anxiety. In both STAI-S and STAI-T, high scores indicate high levels of anxiety. The STAI was adopted to evaluate the divergent validity of the COVID-19-PTSD questionnaire. The reliability of the STAI is adequate (α values ranging between 0.90 and 0.93).

### 2.3. Procedure

A web-based cross-sectional survey, implemented using the Kobo Toolbox platform and broadcasted through the mainstream social-media (such as Facebook, Twitter, Instagram, Telegram), was used to collect data among the Italian speaking population. The survey was enabled from March 18 to 31. A brief presentation informed the participants about the aims of the study, and electronic informed consent was required from each participant before starting the investigation. The completion of all questionnaires took about 30 min. To guarantee anonymity, no personal data, which could allow the identification of respondents, was required. Due to the aim of the current study, the only inclusion criterion was to be at least 18 years of age.

Participants were required to fill in a short demographic questionnaire, and to respond to questions about their knowledge and perceptions of the COVID-19 diffusion. Then, the questionnaires were administered. 

This study was conducted according to the Declaration of Helsinki, and the Ethics Committee of the Department of Dynamic and Clinical Psychology (“Sapienza” the University of Rome, protocol number: 0000266) approved it. Participants could withdraw from the survey at any moment without providing any justification, and no data were saved. 

### 2.4. Statistical Analyses

Three structural models of the COVID-19-PTSD questionnaire were tested using confirmatory factor analysis (CFA). The first CFA tested the monofactorial model, the second evaluated the DSM-5 four-factor model of PTSD, and the third analysis assessed the seven-factors model, as suggested by Ashbaugh et al. [28].

The maximum likelihood (ML) estimation was employed in CFA. Goodness-of-fit was assessed using several fit indices, including Chi-Square, Comparative Fit Index (CFI), Tucker Lewis Index (TLI), Root Mean Square Error of Approximation (RMSEA) and Standardized Root Mean Square Residual (SRMR). The cut-off criteria for the fit indices were based on the guidelines proposed by Hair et al. [36]. Adequate model fit was determined by cut-offs of 0.90 for the CFI and TLI, 0.08 for the RMSEA, and 0.08 for the SRMR. 

In order to compare the three models, chi-square differences and the Akaike information criterion (AIC) were examined. The lowest value of AIC indicates a better comparative fit. 

Cronbach Alpha was calculated for the total COVID-19-PTSD questionnaire and its subscales to assess internal consistency. According to previous studies, the following criteria were accepted for the Cronbach’s alpha: ≥0.9 Excellent; ≥0.8 Good; ≥0.7 Acceptable; ≥0.6 Questionable; ≥0.5 Poor; <0.5 Unacceptable [36,37,38].

Convergent validity was assessed by computing the correlations between the COVID-19-PTSD and the IES—R scores. The z-score differences of the correlations among the COVID-19-PTSD, IES—R and STAI-T were used to assess divergent validity.

Detection analysis, using the ROC curve, allows the defining of the best cut-off score as a predictor of the prevalence of PTSD symptomatology. Despite the modification of the items, the areas investigated by our questionnaire were substantially those of the PCL-5. For this reason, the calculation of the ROC curve was made using the DSM-5 checklist for PCL-5 [28,39]. The checklist was adopted to classify participants into ‘High Symptoms of PTSD’ and ‘Low symptoms of PTSD’. Accordingly, a score equal to or higher than 2, in one of the items of the Intrusion and Avoidance scales and at least two of the items of the Negative Affect and Arousal scales, indicates PTSD risk. If the four scales reached the critical score, the respondent was classified in the group with High Symptoms of PTSD. In the ROC curve, the value with the highest specificity and sensitivity was selected. Moreover, chi-square (*X^2^*) analysis was used to compare the proportion of respondents with high PTSD risk, considering IES—R and COVID-19-PTSD questionnaires.

Pearson’s r correlations were carried out to verify the association between COVID-19-PTSD outcomes, psychological symptomatology and sleep quality, assessed by the SCL-90 and PSQI, respectively. 

SPSS Statistics 24 (IBM corp, Armonk, NY, USA) [40] and R software (Rstudio, PBC, Boston, MA, USA) [41] were used for statistical analyses.

## 3. Results

Participants’ characteristics and the scores of questionnaires are reported respectively in Table 1 and Table 2.

### 3.1. Internal Consistency

The COVID-19 PTSD demonstrated excellent internal consistency of the items (Cronbach’s α = 0.94). Moreover, Cronbach’s alphas for each item of the subscales were also good, considering the DSM-5 four-factors model (Cronbach’s α = 0.70–0.86), and the seven-factors model (Cronbach’s α = 0.52–0.85).

### 3.2. Factor Structure 

The CFA indicated that, for the monofactorial and the DSM-5 four-factors hypotheses, only the SRMR value showed adequate fit (Table 3), while for the seven-factor model, the values of all fit indices reached the appropriate cut-off levels (Table 3). Standardized parameter estimates can be found in Table 4. The factor correlations of the DSM-5 four-factors model (ranging from 0.86 to 0.93) and the seven-factors model (ranging from 0.64 to 0.92) were adequate. The superior fit of the seven-factors model, compared to both the monofactorial (*X^2 =^* 709.43, *p* < 0.0001) and DSM-5′s four-factors model (*X^2 =^* 1852.78; *p* < 0.0001), was highlighted. Finally, the AIC value (113,382) for the seven-factors model was lower than the AIC values of both monofactorial (115,903) and DSM-5 four-factors (115,205) models. These results confirm that the seven-factors model best fits the data. 

### 3.3. Convergent and Divergent Validity 

The correlation between the total score of the COVID-19-PTSD questionnaire and the total score of the IES—R was significant (*r* = 0.70, *p* < 0.0001), suggesting good convergent validity. Moreover, significant positive correlations were observed considering all the IES—R subscales (ranging from 0.39 to 0.66).

The correlation between the COVID-19-PTSD and the STAI-T was significant (*r* = 0.50, *p* < 0.001). However, it was significantly lower than the correlation observed between the COVID-19-PTSD and IES—R scores (the difference between the two correlation coefficients was z = 9.25, *p* < 0.001), supporting good divergent validity.

### 3.4. Detection Analysis

The prevalence of participants with PTSD symptomatology assessed with the DSM-checklist was 27.5%. The ROC curve revealed a COVID-19-PTSD cut-off score of 26. It was the best predictor of PTSD symptomatology with a sensitivity of 0.91 and a specificity of 0.92, and a similar percentage of PTSD symptomatology observed (29.5%; *X*^2^ = 2.24; *p* = 0.13). 

### 3.5. Correlation between COVID-19-PTSD and Psychological Symptomatology 

Significant correlations were found between COVID-19-PTSD scores and the general distress (GSI) measured with SCL-90 (*r =* 0.77, *p* < 0.0001), and sleep disturbance measured with PSQI (*r =* 0.53, *p* < 0.0001). Significantly correlations with the SCL-90 subscales are reported in Table 5.

## 4. Discussion

This study aimed to assess the psychometric proprieties of a new questionnaire designed to investigate the severity of the PTSD symptomatology associated with the current COVID-19 pandemic. A pandemic or epidemic represent traumatic events, which differ in characteristics and diffusion from the traumatic experiences previously analyzed. Secondly, this study intended to examine the prevalence of PTSD symptomatology in the Italian population during the phases immediately following the outbreak of the COVID-19 and the imposed measures of social distancing (in two weeks, from March 18 to 31).

Based on the PCL-5 [16], we developed a questionnaire focused on the assessment of the severity of the symptoms of PTSD related to the current COVID-19 emergency. PTSD symptoms were evaluated according to the DSM-5 criteria.

This study was designed as a consequence of the previous studies about the psychological effect of the COVID-19 pandemic [2,3,4,5,6,42,43]. These studies reported an increased risk of psychopathologies and stress-related disorders, as well as a high rate of PTSD symptomatology [44]. However, although the studies on PTSD are extensive, and in recent years the nature of trauma has been reconsidered [45], questionnaires to analyze medical emergencies protracted over time, such as epidemics and pandemics, have not yet developed. Furthermore, the lack of a validated instrument of rapid administration, for assessing the severity of PTSD symptoms in the Italian population, represents another reason for this study.

Accordingly, we analyze the factorial structure and validity of the COVID-19-PTSD questionnaire, considering different factorial hypotheses. In particular, a monofactorial structure was examined according to the preliminary results of our studies [2,10]. The 4-factors model was assessed according to the DSM-5, which classifies PTSD symptoms into four specific criteria. Finally, a 7-factors structure was tested for its high reliability to cover and describe all the PTSD symptoms [28].

The results proved a good internal consistency and a robust convergent validity of the COVID-19-PTSD questionnaire. The seven-factors model, as suggested by Ashbaugh et al. [28], best fits the data collected by the COVID-19-PTSD, compared to the monofactorial structure or the four-factors structure. The factors evidenced by this model were *Intrusion*, *Avoidance*, *Negative Affect*, *Anhedonia*, *Dysphoric arousal*, *Anxious arousal*, and *Externalizing behavior.*

These results allow the defining of the main symptoms associated with the current worldwide traumatic event, although further studies are needed. The alterations in mood, the intrusions, and the dysphoric and anxious arousal associated with memories of adverse events (i.e., the COVID-19 epidemic) or other related events (e.g., the fear of non-compliance with the restrictive measures requested by the Government) characterized the symptomatology reported by the respondents. The present investigation showed that this self-report questionnaire manages well the assessment of stress conditions related to the trauma. Furthermore, its satisfactory psychometric properties confirm its usefulness, in both the research field and clinical practice.

This study also aimed to identify the prevalence of COVID-19-related PTSD. Previous studies on COVID-19 have reported a prevalence of about 5% of PTSD-related symptoms in Wuhan, the first region of China affected by the COVID-19 epidemic. This result was observed by using the PCL-5, and by considering a cut-off point of 33 [5]. A similar prevalence was reported in the Italian population [10] using the COVID-19-PTSD scores, and considering a cut-off given by the mean score of the overall sample plus 1.5 standard deviations.

In the current study, we applied the PTSD criteria of the DSM-5 to determine the prevalence of PTSD, finding a significantly higher prevalence of PTSD symptomatology [39]. A COVID-19-PTSD score of 26 was deemed to correctly categorize a participant as having or not having significant PTSD symptoms. The prevalence of PTSD, considering this cut-off score, was reported at around 29%. At the first examination, this percentage might seem high. However, different hypotheses can be carried out. Firstly, the data were collected in March 2020, during the peak of infection and death due to COVID-19 in Italy [46], characterized by a high overload of the national health system and the restriction measures adopted by the Italian Government, i.e., a phase that can be considered as a highly acute stressor. Secondly, the specific request to focus on issues related to COVID-19 may have emphasized the results associated with the perception of personal distress, representing a psychological demand from respondents, who experienced for the first time this pandemic, to perceive it as highly traumatic. In this light, the COVID-19-PTSD questionnaire could be considered a sensitive screening tool in a population faced with emergencies, and it can represent a first step toward the assessment of the risk of PTSD. However, further studies are needed because of the absence of clinical guidelines to define a clear cut-off for the diagnosis of PTSD, and given the lack of studies adopting this instrument on other samples.

The COVID-19-PTSD questionnaire scores were positively correlated with the psychopathological symptoms assessed by the SCL-90, and the sleep disturbance evaluated by the PSQI. These findings confirm the association between general psychopathological symptomatology and the COVID-19 emergency, as shown by previous studies that have reported on the Italian population’s sleep disturbance, state of anxiety, altered mood and psychological distress related to the COVID-19 condition [2,15,47].

These results appear essential to defining a possible therapeutic approach during, and at the end of, this emergency. The COVID-19 pandemic, unexpectedly and suddenly, has generated fear, has limited individual freedom, and it has led to a distortion of emotional and social relationships. All these aspects should be taken into account to avoid the development or the worsening of PTSD symptomatology.

Although the results of this study are promising, some limitations emerged. A first limitation is the same as that suggested by Ashbaugh et al. [28], about the presence of factors reporting only two items, which could influence their reliability. However, considering our seven-factors model, except for the Externalizing Behavior, the other factors reported adequate consistency indices (Cronbach’s α ≥ 0.70) [48]. Another limit is represented by the absence of a test–retest analysis, considering a translation of the classic version of the PCL-5 not directly referred to the COVID-19 experience. A similar evaluation could help us in determining similarities and differences between the two questionnaires, allowing us to confirm what assessment represents the better choice in this emergency. Moreover, despite the large sample size, it is not possible to overcome the limitations of a cross-sectional online study, such as the selection bias of participants’ recruitment and the absence of a longitudinal control aimed to identify the real risk of the PTSD occurrence. This bias is expressed by the higher number of respondents younger than 30 years of age, and the higher number of females and people from South Italy. Finally, it could be useful to replicate these results in a clinical sample. Furthermore, it could be helpful in other studies to perform multilevel analyses (e.g., considering age, gender or geographical location) to differentiate PTSD symptoms. Surely, these limitations reduce the representativeness of our findings, and may have influenced the results of the study. However, the adoption of an online survey was the best solution in this emergency, in which social distancing measures have limited data collection.

## 5. Conclusions

This study is the first to propose an Italian-language version of a questionnaire assessing PTSD symptoms, referring to the COVID-19 pandemic as a traumatic event. This questionnaire demonstrated excellent reliability, as well as convergent validity. Using CFA, our data showed a better fit with a seven-factors model, compared to the four-factors model based on the DSM 5 criteria and a monofactorial model. The focus on the assessment of the specific stress-related events of the COVID-19 pandemic, the quarantine measures, and their impact on individual life, makes this study very important from a clinical point of view. The COVID-19-PTSD questionnaire appears to be useful for measuring symptom severity of PTSD in the Italian population.

In addition to providing the psychometric properties of this self-report measure, our study evidenced a significant prevalence of symptomatology related to the COVID-19 emergency in the Italian population. This event, with its unique characteristics, could generate a novel type of trauma, substantiated by the sum of numerous factors (i.e., the fear of contracting the infection, social distancing, the restriction of one’s individual choices, the change in lifestyle and interpersonal relationships, economic problems, uncertainty about the future, etc.). Therefore, further studies are useful in this field. We hope that these results could serve as a guide for other studies on this pandemic, but we also hope to validate our questionnaire with further clinical samples in different medical emergencies. Finally, focusing on the risk of PTSD in this dramatic moment for the Italian population (and the world population) can lead to the development of effective prevention programs and therapeutic interventions.

## Figures and Tables

**Table 1:** Demographic characteristics of the sample.

Variables	Total Sample (*N* = 2286)
*Sex, n (%)*	
Man	580 (25.4)
Woman	1706 (74.6)
*Age, n (%)*	
18–29 years old	1568 (68.6)
30–49 years old	485 (21.2)
>50 years old	233 (10.2)
*Education, n (%)*	
Until middle School	97 (4.2)
High School	1135 (49.6)
*Undergraduate*	
Health care *	246 (10.8)
Other	658 (28.8)
*Post-graduated*	
Health care	63 (2.8)
Other	87 (3.8)
*Occupation, n (%)*	
Student	1071 (46.8)
Employed	687 (30.1)
Unemployed	278 (12.2)
Self-Employed	222 (9.7)
Retired	28 (1.22)
*Territorial Areas*	
North Italy	540 (23.6)
Centre Italy	571 (25.0)
South Italy	1175 (51.4)
*Number of inhabitants in own city, n (%)*	
<2.000	124 (5.4)
2.000–10.000	451 (19.7)
10.000–100.000	936 (50.0)
>100.000	775 (33.9)
*Psychopathological History*	
Yes	113 (5.0)
No	2173 (95.0)
*Quarantine Experience, n (%)*	
Alone	2054 (89.9)
Others	232 (10.1)
*Infection by the virus*	
Yes	9 (0.4)
No	1703 (74.5)
Do not know	574 (25.1)
*Direct contact with people infected by COVID-19*	
Yes	40 (1.7)
No	1438 (63.0)
Do not know	808 (35.3)
*Knowledge of people infected by COVID-19*	
Yes	549 (24.0)
No	1737 (76.0)
*Knowledge of people in ICU for COVID-19*	
Yes	177 (7.7)
No	2109 (92.3)
*Knowledge of people died for COVID-19*	
Yes	112 (4.9)
No	2174 (95.1)

ICU: Intensive Care Unit. * Health care refers to nursing, medical doctor, psychologist, pharmacologist, dentistry, etc.

**Table 2:** Mean, range of observed values, and standard deviation of the variables.

Variables	Means (Range)	Standard Deviation
COVID-19-PTSD		
Total Score	19.87 (0–76.0)	15.88
Intrusion	4.26 (0–16.0)	3.76
Avoidance	2.48 (0–8.0)	2.12
Negative Mood	2.83 (0–12.0)	2.95
Anhedonia	3.23 (0–12.0)	3.09
Externalizing behavior	1.19 (0–8.0)	1.59
Anxious Arousal	2.54 (0–8.0)	2.27
Dysphoric Arousal	3.36 (0–12.0)	3.49
IES—R		
Total Score	22.38 (0–88.0)	18.10
Intrusion	1.01 (0–4.0)	0.91
Avoidance	1.06 (0–4.0)	0.83
Iperarousal	0.98 (0–4.0)	0.93
SCL-90		
Somatization	0.71 (0–4)	0.71
Obsessive-Compulsive	0.91 (0–4)	0.91
Interpersonal Sensitivity	0.58 (0–3.9)	0.58
Depression	1.01 (0–3.6)	1.01
Anxiety	0.86 (0–3.8)	0.75
Anger-Hostility	0.65 (0–4)	0.64
Phobic Anxiety	0.59 (0–3.7)	0.71
Paranoid Ideation	0.57 (0–3.8)	0.65
Psychoticism	0.49 (0–3.3)	0.53
Sleep Disturbance	0.37 (0–1.3)	0.36
Global Index	0.74 (0–3.3)	0.59
PSQI		
Total Sleep Disturbance	5.69 (0–20.0)	3.40

PTSD: Post-Traumatic Stress Disorder; IES-R: Impact of Event Scale—Revised; SCL-90: Symptom Checklist-90; PSQI: Pittsburgh Sleep Quality Index.

**Table 3:** Confirmatory factor analyses results for Monofactorial Model, DSM-5 4-factors model and 7-factors model.

Fit Indices	Monofactorial Model	DSM-5 4-Factor Model	7-Factor Model
X^2^/df	29.31 *	25.65 *	14.45 *
CFI ^1^	0.84	0.86	0.93
TLI ^2^	0.82	0.84	0.91
RMSEA (CI 95%) ^3^	0.11 (0.108–0.114)	0.10 (0.101–0.107)	0.07 (0.072–0.079)
SRMR	0.06	0.05	0.06

^1^ Comparative Fit Index (cut-off ≥ 0.90). ^2^ Tucker-Lewis Index (cut-off ≥ 0.90). ^3^ Root Mean Square Error of Approximation (cut-off < 0.08).^4^ Standardized root mean square (cut-off ≤ 0.08). * *p* < 0.0001.

**Table 4:** The estimated parameters of the factor models of Confermatory Factor Analysis. The items are shown in Italian, and the English translation is shown in parentheses.

COVID-19-PTSD Items	Monofactorial Model		DSM-5 4-Factor Model		7-Factor Model	
	Factor	Factor Loading	Factor	Factor Loading	Factor	Factor Loading
1. Avere pensieri ripetuti, inquietanti e indesiderati relativi a questa esperienza stressante (Having repeated, disturbing and unwanted thoughts related to this stressful experience)	1	0.78	1	0.84	1	0.84
2. Avere sogni ripetuti e inquietanti relativi a questa esperienza stressante (Having repeated and disturbing dreams related to this stressful experience)	1	0.57	1	0.59	1	0.58
3. Sentirsi molto turbato (Feeling very upset)	1	0.80	1	0.86	1	0.86
4. Avere forti reazioni fisiche pensando a questa esperienza stressante (es. cuore martellante, difficoltà a respirare) [Having strong physical reactions thinking about this stressful experience (e.g., pounding heart, difficulty breathing)]	1	0.67	1	0.69	1	0.69
5. Cercare di evitare pensieri e sentimenti legati a questa esperienza stressante (Try to avoid thoughts and feelings related to this stressful experience)	1	0.58	2	0.62	2	0.63
6. Avere difficoltà a pensare ad aspetti diversi da questa situazione stressante (Having difficulty thinking about aspects other than this stressful situation)	1	0.78	2	0.85	2	0.85
7. Avere forti convinzioni negative su te stesso/a, gli altri o il mondo (es. avere pensieri come: sto male, qualcuno a me caro si sta ammalando, il mondo è diventato pericoloso) [Having strong negative beliefs about yourself, others or the world (e.g., having thoughts like: I’m sick, someone dear to me is getting sick, the world has become dangerous)]	1	0.75	3	0.76	3	0.80
8. Incolpare te stesso/a o qualcun altro per non aver adottato comportamenti adeguati alla situazione (es., sono andato/a al Pub, al ristorante, ecc.) [Blaming yourself or someone else for failing to adopt appropriate behaviors to the situation (eg., I went pub, restaurant, etc.)].	1	0.50	3	0.53	3	0.55
9. Avere forti sentimenti negativi come paura, orrore, rabbia, colpa o vergogna (Having strong negative feelings like fear, horror, anger, guilt or shame)	1	0.78	3	0.80	3	0.84
10. Avere perdita di interesse per le attività che ti piacevano (Experience loss of interest in the activities you liked)	1	0.67	3	0.70	4	0.75
11. Sentirti distante dalle altre persone (Feeling distant from other people)	1	0.54	3	0.58	4	0.66
12. Avere difficoltà a provare sentimenti positivi (es., essere incapace di provare felicità o affetto verso le persone vicino a te) [Having difficulty to feel positive feelings (e.g., being unable to feel happiness or positive affects for people close to you]	1	0.70	3	0.74	4	0.84
13. Avere un comportamento irritabile, esplosioni di rabbia o azioni aggressive (Having irritable behavior, outbursts of anger or aggressive actions)	1	0.64	4	0.67	5	0.77
14. Assumere troppi rischi o fare cose che avrebbero potuto metterti a rischio (Taking too many risks or doing things that could put you at risk)	1	0.43	4	0.44	5	0.49
15. Essere ipervigili rispetto alla condizione attuale (Being hypervigilant over the current condition)	1	0.66	4	0.66	6	0.68
16. Sentirti nervoso/a o facilmente spaventato/a (To feel to be nervous or easily frightened)	1	0.85	4	0.86	6	0.88
17. Avere difficoltà a concentrarti (To have trouble concentrating)	1	0.73	4	0.76	7	0.66
18. Avere problemi ad addormentarti (To have difficulty in falling asleep)	1	0.65	4	0.68	7	0.90
19. Avere un sonno disturbato (To have a disturbed sleep)	1	0.66	4	0.69	7	0.91

**Table 5:** Correlations among the scales of the 7-factors model of the COVID-19-PTSD and the outcomes of SCL-90 and PSQI.

	Total Score	Intrusion	Avoidance	Negative Mood	Anhedonia	Externalizing Behavior	Anxious Arousal	Dysphoric Arousal
**SCL-90**								
Somatization	0.61	0.55	0.46	0.49	0.49	0.43	0.50	0.55
Obsessive-Compulsive	0.66	0.51	0.50	0.53	0.60	0.51	0.51	0.62
Interpersonal Sensitivity	0.54	0.38	0.38	0.46	0.54	0.51	0.41	0.46
Depression	0.74	0.59	0.57	0.59	0.70	0.54	0.58	0.64
Anxiety	0.77	0.70	0.59	0.65	0.60	0.54	0.67	0.65
Anger-Hostility	0.57	0.40	0.39	0.46	0.54	0.66	0.46	0.48
Phobic Anxiety	0.58	0.55	0.44	0.54	0.41	0.39	0.55	0.43
Paranoid Ideation	0.48	0.37	0.33	0.43	0.47	0.48	0.37	0.38
Psychoticism	0.59	0.45	0.41	0.51	0.58	0.52	0.44	0.49
Sleep Disturbance	0.63	0.51	0.44	0.45	0.48	0.42	0.48	0.74
Global Index	0.77	0.63	0.57	0.64	0.68	0.60	0.62	0.67
**PSQI**								
Total Sleep Disturbance	0.53	0.44	0.36	0.38	0.39	0.30	0.40	0.70

Significance: *p* < 0.0001.

## Supplementary Materials

The following are available online at https://www.mdpi.com/1660-4601/17/11/4151/s1, Table S1: The COVID-19-PTSD Questionnaire.
