# Supplementary material for: COVID-19 Pandemic in the Italian Population: Validation of a Post-Traumatic Stress Disorder Questionnaire and Prevalence of PTSD Symptomatology
Source: Int J Environ Res Public Health. 2020 Jun 10;17(11):4151. doi: 10.3390/ijerph17114151 (PMC7312976; doi:10.3390/ijerph17114151)
Supplement: Supplementary file 1 [file ijerph-17-04151-s001.pdf]

Supplementary Material. The COVID-19-PTSD Questionnaire.

Facendo riferimento alla situazione attuale, caratterizzata dall'epidemia di COVID-19 e dalle misure di distanziamento sociale messe in atto per contenerla, indichi come si sente per ciascuna delle seguenti dimensioni.

	**Per niente**	**Un pò**	**Moderatamente**	**Abbastanza**	**Estremamente**
1. Avere pensieri ripetuti, inquietanti e indesiderati relativi a questa esperienza stressante					
2. Avere sogni ripetuti e inquietanti relativi a questa esperienza stressante					
3. Sentirsi molto turbato					
4. Avere forti reazioni fisiche pensando a questa esperienza stressante (es. cuore martellante, difficoltà a respirare)					
5. Cercare di evitare pensieri e sentimenti legati a questa esperienza stressante					
6. Avere difficoltà a pensare ad aspetti diversi da questa situazione stressante					
7. Avere forti convinzioni negative su te stesso/a, gli altri o il mondo (es. avere pensieri come: sto male, qualcuno a me caro si sta ammalando, il mondo è diventato pericoloso)					
8. Incolpare te stesso/a o qualcun altro per non aver adottato comportamenti adeguati alla situazione (es., sono andato/a al Pub, al ristorante, ecc.)					
9. Avere forti sentimenti negativi come paura, orrore, rabbia, colpa o vergogna					
10. Avere perdita di interesse per le attività che ti piacevano					
11. Sentirti distante dalle altre persone					
12. Avere difficoltà a provare sentimenti positivi (es., essere incapace di provare felicità o affetto verso le persone vicino a te)					
13. Avere un comportamento irritabile, esplosioni di rabbia o azioni aggressive					
14. Assumere troppi rischi o fare cose che avrebbero potuto metterti a rischio					
15. Essere ipervigili rispetto alla condizione attuale					
16. Sentirti nervoso/a o facilmente spaventato/a					
17. Avere difficoltà a concentrarti					
18. Avere problemi ad addormentarti					
19. Avere un sonno disturbato					
